# The Serum Treatment of Yellow Fever

**Published:** 1897-10-30

**Authors:** 


### THE SERUM TREATMENT OF YELLOW FEVER.

We must await the full report of Professor Sanarelli's lecture at Monte Video on the "Serum-therapy of Yellow Fever" before we can indulge in any very confident hope that a successful treatment for this very fatal disease has been discovered. There is no doubt that a certain bacillus has been isolated, and that by the usual process the serum-giver has been rendered immune to considerable doses of this bacillus, and it appears that the serum yielded by this animal has conferred on the guinea-pigs injected with it a certain degree of immunity to "experimental yellow fever," in other words, to the disease produced by inoculation with the bacillus in question, and has even succeeded in saving the lives of guinea-pigs already ill of this disease.

The question still remains, however, whether this experimental yellow fever is the same disease as the real thing, and we must hope that Professor Sanarelli will be able to give much stronger proof than has hitherto been afforded regarding the activity of his serum and of the identity of "experimental" with real yellow fever.

